# New oral anticoagulants: are coagulation units still required?

**DOI:** 10.1186/1477-9560-12-3

**Published:** 2014-02-03

**Authors:** Raul Altman

**Affiliations:** 1 Centro de Trombosis Buenos Aires, Viamonte 2008, Buenos Aires 1056, Argentina

**Keywords:** New oral anticoagulant, NOAC, Bleeding, Laboratory control

## Abstract

Chronic antithrombotic therapy involves the use of anticoagulants, antiplatelets given either as monotherapy or in combination for the prevention of thrombotic complications. The most feared and sometimes fatal complication with this therapy is bleeding. It should be considered a “golden rule” that a drug or combination of drugs that maximizes efficiency (decreased thromboembolic risk) will probably be less safe (increased risk of bleeding), and this holds true either for single therapy or during combined therapy. The chances of bleeding indicated by risk tables can be useful but show only a snapshot, and the biological, social, environmental, and drug changes and therapeutic adherence also determine changes in the risk of thrombosis and bleeding. Bleeding is an eventuality that occurs in places of “locus minoris resistentiae,” and the results of careful phase 3 studies thus cannot be completely predictive of outcomes when a medication is introduced on the pharmaceutical market. With the use of warfarin, the International Normalized Ratio (INR) that has been established to indicate adequately balanced therapy is between 2.0 and 3.0. With the new oral anticoagulants, the pharmaceutical companies emphasize that it is not necessary to monitor anticoagulant effects. In studies with different doses of new oral anticoagulants, however, incidence of clinically significant bleeding complications have been directly related to the doses. Therefore, therapeutic excesses can condition bleeding risk and therapeutic limitation can increase thrombotic risk, especially when short-acting drugs such as the new oral anticoagulants are used. Hence, it is imperative to establish an appropriate method for monitoring new oral anticoagulants, setting levels of safety and effectiveness through periodic dosage and monitoring of their anticoagulant effects. Therefore, we still recommend the use of anticoagulation units for monitoring during treatment with the new oral anticoagulants.

## Introduction

Chronic antithrombotic therapy involves the use of anticoagulants, antiplatelets that are given either as monotherapy or in combination for the prevention of thrombotic complications. The well-established benefits of anticoagulant therapy are significantly hampered by the possibility of major and sometimes fatal bleeding complications. These adverse effects can range from simple skin bruising and bleeding bodies in relation to the outside (epistaxis, gastroduodenal bleeding, pulmonary complications) [1,2] or can affect vital organs with temporary or permanent impairment of function (intracranial hemorrhage) or death.

Another risk of even mild bleeding complications is that they quite frequently lead to the discontinuation of the anticoagulant therapy, either by the patient or the physician. The loss of protection by the cessation of the antithrombotic treatment obviously will favor the development of thromboembolic complications [3].

Until very recently, warfarin was the only oral anticoagulant medication available, and undoubtedly it was and still is considered the “gold standard” for the prevention of ischemic stroke in patients with atrial fibrillation (AF) and/or cardiac valve prosthesis in deep vein thrombosis and pulmonary embolism. Its efficacy has been well-established by several large clinical trials comparing warfarin versus placebo or antiplatelet agents [4]. This proven efficacy is seriously reduced, however, by several limitations that affect its wide clinical use [5,6]. Among the major limitations of treatment with vitamin K antagonists are a narrow therapeutic window, slow onset and offset of action, numerous interactions with food and drugs, and an unpredictable response that requires a systematic monitoring of anticoagulation and frequent changes in dose.

The increasing aging of the world population may be a partial explanation for the increase in the rates of AF diagnosis and other pathological conditions requiring chronic anticoagulation. These limitations and the increase in numbers of AF patients have triggered an active search for better and/or safer anticoagulant drugs.

The new US Food and Drug Administration–approved oral anticoagulants dabigatran, rivaroxaban, and apixaban are administered as a fixed-oral daily dose to all patients and seem to share some advantages. In fact, the new oral anticoagulants work at different levels of the tissue factor (TF) pathway and could be divided, based on their mechanism of action, into two major classes: i) direct thrombin inhibitors (dabigatran) and ii) inhibitors of the activated coagulation factor X (rivaroxaban and apixaban).

Dabigatran etexilate is the prodrug of dabigatran that reversibly inhibits the thrombin active sites of both free thrombin and thrombin-bound to fibrin. About 80% is excreted unchanged by the kidney so its administration is contraindicated in patients with renal failure. Rivaroxaban is a small molecule with direct inhibitory activity on activated factor X. It is rapidly absorbed and has a high bioavailability, is administered once daily, and has a very short half-life of 5–9 h in healthy volunteers but that is significantly higher in the elderly (9–13 h). It is eliminated by the kidneys and liver. Apixaban is a potent and selective inhibitor of activated factor X with a half-life of 12 h, 60% bioavailability, and elimination by multiple routes including via the hepatic metabolism by cytochrome P450 3A4. In various dose-finding studies with graded doses of this new oral anticoagulant, the incidence of clinically significant bleeding complications was directly related to the dose (Table 1) [7-10].

**Table 1:** Bleeding events with new oral anticoagulants in dose-finding studies

**Study drug: endpoint, bleeding**	**Daily dose**			
***Dabigatran*** [8]	*50 mg × 2*	*150 mg × 2*	*300 mg*	*225 mg × 2*
Clinically important %	2.3	4.1	4.9	5.1
***Rivaroxaban*** [9,10]	*5 mg*	*10 mg*	*20 mg*	*30 mg*
Major after surgery %	2.2	2.3	4.5	5.4
***Edoxaban*** [11]	*30 mg*	*30 mg × 2*	*60*	*60 mg × 2*
Major + clinically relevant %	3.0	7.8	3.8	10.6

Several major clinical trials have compared the new oral anticoagulants with warfarin in AF patients (RELY, ROCKET-AF, and ARISTOTLE) [11-13]. Because of the better or non-inferior efficacy and lower risk of bleeding complications combined with fewer interactions with other drugs and food, the new oral anticoagulants are emerging as a viable alternative to warfarin for the prevention of stroke and thromboembolic complications in AF patients.

Therefore, it could be postulated that the new oral anticoagulants, when combined with acetylsalicylic acid (ASA) or the new PY2 inhibitors in acute coronary syndrome patients, may offer some therapeutic benefit over the traditional warfarin and aspirin. At the same time, however, it might bring some increases in bleeding complications within the “golden rule” in thrombosis that those interventions that maximize efficiency (reduction of events) will probably also increase bleeding risk. It is therefore necessary to have an idea of the condition of the patient and to establish the bleeding risk, perhaps through risk tables. Bleeding is an eventuality that occurs in places of “locus minoris resistentiae,” however, and the results of careful phase 3 studies thus cannot be considered fully predictive of “real life” when a medication enters the pharmaceutical market.

### Monotherapy

The use of coumarin as an anticoagulant forced the establishment of a reference for controlling plasma therapeutic level. The introduction of International Normalized Ratio (INR) offered an appropriate method to assess this anticoagulant effect. It is widely accepted that the optimal therapeutic level when using coumarin should be within INR values of 2.0 and 3.0, or between 2.0 and 3.5 in some circumstances [14,15]. Lower values will not offer protection while higher values promote bleeding risk, especially in individuals of advanced age [16].

Eikelboom et al. used dabigatran in patients with mechanical heart valves. The initial dabigatran dose (150, 220, or 300 mg twice daily) was based on kidney function and was adjusted to obtain a trough plasma level of at least 50 ng/ml, but no clotting tests were used to adjust therapy. The trial was terminated prematurely after the enrollment of 252 patients because of an excess of thromboembolic and bleeding events among patients in the dabigatran group [17].

### Combined therapy

Combining two antiplatelet drugs or oral anticoagulant and one or two antiplatelet drugs produces an increase of bleeding. Hansen et al. [18] found in patients with AF that combinations of warfarin, aspirin, or clopidogrel were associated with an increased risk of fatal and non-fatal bleeding. In a meta-analysis involving 5938 patients who had suffered acute coronary syndrome, the addition of warfarin to aspirin significantly decreased thrombotic events vs. aspirin alone, but resulted in higher major bleedings [19]. Another study compared ASA vs ASA + clopidogrel in warfarin-intolerant patients with AF, and the combination therapy reduced major ischemic events, particularly stroke, but at the expense of an increase in major bleeding (2% per year vs. 1.3% per year; p = 0.001) [20]. Similar results were obtained in patients with lacunar stroke (SPS3 study); the addition of clopidogrel to aspirin did not significantly reduce recurrent stroke but significantly increased the risk of bleeding and mortality [21].

Of importance, in real life, not only the major or minor bleeding but also skin hemorrhages lead patients to discontinue therapy and thus increase the potential risk of thrombosis. Chronic administration of a combination of antiplatelets has been associated with a high level of nuisance hemorrhages [22]. Antiplatelet combinations significantly prolong bleeding time and increase the incidence of subcutaneous hematomas, epistaxis, hematuria, and severe proctorrhagia [23].

A similar observation seems to apply to the new oral anticoagulants as per the APPRAISE-2 trial. The addition of apixaban to antiplatelet therapy in high-risk acute coronary syndrome patients did not significantly reduce ischemic events but did increase the number of major bleedings, including intracranial and fatal hemorrhage [24].

Unfortunately, the need is not uncommon for antiplatelet (ASA + clopidogrel) and an anticoagulant combination in patients with heart valve prosthesis, AF, and acute coronary events or coronary stenting. In these pathological conditions, the theoretical advantage of thrombotic prevention should be considered against the risk of bleeding [25,26].

Patients on warfarin undergoing stenting should receive dual antiplatelet therapy in addition to anticoagulation. This scenario was studied by Woest et al., who compared warfarin + clopidogrel vs. warfarin + clopidogrel + aspirin. The results showed improved endpoints with the triple medication but also an increased bleeding that exceeded efficacy (Table 2) [25].

**Table 2:** **Efficacy and bleeding in patients treated with oral anticoagulant + clopidogrel (double therapy) or oral anticoagulant + clopidogrel + aspirin (triple therapy) (data modified from **[19]**)**

**Secondary and safety endpoints at 1 year**	**Double therapeutic %**	**Triple therapeutic %**	**n**	**NNT**
Total efficacy	11.1	17.6	0.025	15
Deaths (all causes)	2.5	6.5	0.027	25
Myocardial infarction	3.2	4.6	0.328	71
Stroke	1.1	2.5	0.128	71
Stent thrombosis	1.4	3.2	0.125	56
Any bleeding event	19.4	44.4	<0.001	4.0

NNT: Number need to treat.

It has been postulated that in those situations requiring warfarin and dual antiplatelet therapy, the level of anticoagulation should be lowered to an INR of 2.0–2.5 to reduce bleeding risks without affecting efficacy [27]. This rule cannot be applied to the new oral anticoagulants because of the lack of an effective and reliable clotting assay to measure anticoagulation when using the new agents.

The benefits of the new oral anticoagulants in acute coronary syndrome patients receiving antiplatelet therapy were investigated in a meta-analysis of seven prospective studies involving 31,286 patients. The use of direct thrombin inhibitors or FXa inhibitors was associated with significant increases in major bleeding that exceeded the benefits for patients receiving single antiplatelet therapy [28]. Similar conclusions were reached in a more recent meta-analysis [29].

### Why patients bleed? “The Golden Rule” in antithrombotic therapy

An abundance of literature supports what we have called “The Golden Rule” in antithrombotic therapy. Bleeding charts such as the HAS-BLED have been proposed to estimate the bleeding risk, including intracranial bleeding in AF patients on anticoagulant therapy with warfarin [30-34]. The HAS-BLED score includes hypertension, renal conditions, and/or liver disease, history of stroke, bleeding, the INR lability, age over 65 years, and alcohol or drug intake [31]. Despite its popularity, the clinical significance of 7 scoring systems for risk of bleeding events in patients on anticoagulant therapy was poor and no better than the subjective evaluation of a physician [35].

As described before, among the limitations of warfarin, biological, social, and environmental factors, emerging medical conditions, and adherence to treatment could vary and also determine changes in the risk of bleeding. These variations justify the need for tight medical control of warfarin-treated patients. But is the scenario different when using the new oral anticoagulants? Because of their short half-life in combination with the assumption that they do not require routine coagulation monitoring, patient adherence becomes an extremely important issue [36].

The full benefit of biologic therapies is not reached and quality of life is compromised if patients do not adhere to their medication regimen, and poor adherence is a leading cause of preventable morbidity and mortality. Among reasons for non-adherence are dislike of taking medications on a long-term basis and uncertainty about the need for treatment. The consequences of poor medication adherence contribute to 33 to 69% of hospital admissions in the United States and cause up to 40% of admissions to US nursing homes [37]. The use of potentially inappropriate medications in older people is associated with an increased risk of adverse drug events and hospitalization [38].

The mostly hepatic metabolization via the cytochrome P450 pathway seems responsible for the high degree of interaction with food and other medications of the Vitamin K antagonists. P-glycoprotein (P-gp) is involved in the transport of many drugs such as the new oral anticoagulants and is another potential reason for drug interaction. Therefore, agonists or inhibitors of these pathways could affect potential drug interactions with these new therapies [39,40].

The plasma concentration of a new oral anticoagulant will increase or decrease upon the concomitant presence of potent P-gp inhibitors or agonists, respectively. In either case, alterations of new oral anticoagulant pharmacokinetics upon interactions of CYP3A4 or P-gp may complicate the use of these compounds in daily practice, suggesting the appropriateness of controlling anticoagulant activity for improving efficacy and safety.

Dabigatran requires caution when used in combination with strong inhibitors or inducers of P-gp, such as amiodarone or rifampicin. Quinidine used together with dabigatran also is contraindicated. Rivaroxaban (and possibly apixaban) is contraindicated in combination with drugs that strongly inhibit both cytochrome P450 3A4 and P-gp, such as azole antimycotics, and caution is required with its use in combination with strong inhibitors of only one of these pathways [41]. Important drug interactions of the new oral anticoagulants that can lead to adverse clinical reactions may also occur with non-steroidal anti-inflammatory drugs and antiplatelet drugs, such as aspirin and clopidogrel. Clinical experience regarding food interactions is currently limited [41]. Table 3 shows some drugs that induce P-gp activity and P-gp inhibitors that could modify plasma levels of the new oral anticoagulants and their anticoagulant activities.

**Table 3:** Some inducers and inhibitors of P-glycoprotein frequently used in medicine

**Inducers**	**Inhibitors**
Bromocriptine, carbamazepine, colchicine, cyclosporine, dexamethasone, indinavir, morphine, rifampicin, St. John’s wort, tacrolimus, phenytoin, phenobarbital	Amiodarone, amitriptyline, astemizole, carvedilol, clarithromycin, cortisol, diltiazem, disulfiram, erythromycin, fluoperazine, arandalos juice, haloperidol, nifedipine, quinidine, ritonavir, simvastatin, testosterone, tacrolimus, verapamil

Many drug interactions have shown the involvement of P-gp and CYP3A4 due to the overlap of substrate specificity and the similarities in their inducers and inhibitors. Thus, it can be assumed that the theoretical possibilities are not fully understood when the new oral anticoagulants are administered to patients, and controlling their anticoagulant effect is necessary or mandatory or at the least, highly desirable (in acute bleeding, prior to surgery or invasive procedures, in the possibility of overdose or subtherapeutic dose, in patients with moderate to severe renal insufficiency or middle or severe hepatic damage, for testing drug interaction, in elderly patient, in obese or very thin patient, or for checking treatment adherence). This stance is strongly supported by Bloemen et al. [42], who demonstrated that the addition of a fixed concentration of any type of anticoagulant causes a highly variable inhibition of thrombin generation in different individuals.

Hemostasis is a local process and occurs normally in minutes, and thrombin formation is limited [43]. Furie and Furie mentioned that after injury, the formation of fibrin is derived from the thrombin generated by the TF exposed from the vessel wall [44]. The process is circumscribed because the amount of exposed TF is small in a limited area, and activators are diluted in the bloodstream in a non-occluded vessel.

In contrast, arterial thrombosis occurs in areas of atherosclerotic plaque disruption. Atherosclerotic lesion disruption facilitates the interaction of the circulating blood with the inner components of the lesions, such as TF, and this interaction leads to the in vivo generation of thrombin. The stasis in a partially or fully occluded vessel determines the local increase of thrombin, both free and bound to fibrin. The flow-limiting effect of the disrupted lesion prevents dilution of the activators and favors the formation of larger or more stable thrombi.

One of the purposes of antithrombotic therapy is to inhibit the pro-thrombotic activity of thrombin, either by interfering with its synthesis via inhibition of factor Xa (rivaroxaban, apixaban) or directly blocking its activity (dabigatran). The relatively small amount of thrombin that is formed in the case of hemostasis compared with thrombosis will be strongly affected by antithrombotic drugs with impaired hemostasis, and the potential consequence may be bleeding in a “locus minoris resistentiae” [45]. Although major bleeding could be a reversible event, it is likely to lead clinicians to discontinue antithrombotic therapy, which in turn could increase the risk of myocardial infarction, stroke, and cardiovascular death. Therefore, therapeutic excesses can condition bleeding risk, and therapeutic limitation can increase thrombotic risk, especially when short-acting drugs such as the new oral anticoagulants are used. Hence, it is imperative to establish an appropriate method for monitoring new oral anticoagulants and to set the levels of safety and effectiveness by monitoring their anticoagulant effects.

## Competing interests

The author declares that he has no competing interests.
